# Traumatic Brain Injury Induces Early Barrier Protective Responses in Incisional Skin Wounds Accelerating Cutaneous Wound Healing

**DOI:** 10.1111/wrr.70079

**Published:** 2025-08-29

**Authors:** Mahyar Aghapour, Florian Olde Heuvel, Albrecht Fröhlich, Adelheid Heinzl, Pallab Maity, Karmveer Singh, Yongfang Wang, Jinnan Cheng, Francesco Roselli, Karin Scharffetter‐Kochanek

**Affiliations:** ^1^ Department of Dermatology and Allergic Diseases Ulm University Ulm Germany; ^2^ Department of Neurology Ulm University Ulm Germany

**Keywords:** skin barriers, traumatic brain injury, wound healinginnate and adaptive immunity

## Abstract

Though Traumatic Brain Injury (TBI) and skin trauma often occur together, it is unresolved whether TBI changes the healing of skin wounds. We here explored whether TBI impacts the sequence of events during skin wound healing. Incisional skin wounds from mice subjected to TBI were assessed employing unbiased transcriptome analysis and immunostaining. Transcriptome analysis at day 1 after combined trauma detects a significant enrichment of genes involved in macrophage and T cell recruitment and activation in contrast to skin wounds without TBI. At day 7 after combined trauma, genes in pathways of re‐epithelialisation including cornification and keratinisation and of anti‐inflammatory responses were highly enriched. These findings were confirmed by immunostaining with increased re‐epithelialisation and cornification and an increased number of macrophages and T cells resolving inflammation. Moreover, the number of dermal myofibroblasts is highly increased in skin wounds after combined trauma. Collectively, TBI induces a robust defence response characterised by early onset of enhanced immunity, faster epidermal barrier formation, and myofibroblast‐driven acceleration of wound closure, which may together help counteract systemic infection.

## 1 Introduction

Traumatic brain injury (TBI) is a neurological disorder that not only alters brain functionality but in many ways also negatively affects peripheral organs [1]. TBI perpetuates systemic inflammation and exerts profound modifications of the adrenergic/cholinergic autonomic system, leading to barrier disruption of the lung and the gastrointestinal tract among other adverse effects [2]. Nevertheless, it is unknown whether and how TBI affects different wound healing phases in skin injury co‐occurring with brain trauma often observed in accidents. Wound healing of the skin is essential to restore its functionality as a barrier against infection. Skin wounds that do not heal are a constant threat for systemic infections and deadly sepsis. This is particularly true for TBI, which is responsible for severe immune suppression [3] and high susceptibility for infections. In fact, many patients with TBI die from a systemic infection [4]. It is therefore of high clinical relevance to advance our insight into wound healing of the skin after combined TBI and skin trauma.

Currently, it is unclear whether and how TBI affects healing of skin injury, often co‐occurring with brain trauma in accidents. Physiologically, wound healing of the skin is initiated by the phase of haemostasis in which platelets and platelet‐derived factors form a first clot to primarily stop bleeding at the site of injury. Clotting is followed by the early inflammation phase with recruitment of neutrophils and pro‐inflammatory macrophages to mitigate pathogen invasion and propagation. Pro‐inflammatory macrophages clean the wounds by engulfing apoptotic and necroptotic neutrophils beside bacteria. Thereafter, in the late inflammation phase, pro‐regenerative, arginine synthase expressing macrophages resolve inflammation, instruct myofibroblasts to deposit interstitial collagens and foster myofibroblast‐dependent wound contraction. In parallel, epidermal barrier formation occurs to additionally counteract microbial invasion [5, 6].

Previously unreported, we have discovered that TBI improves wound healing of the skin by accelerating the early phase of immune cell recruitment, and subsequent induction of anti‐inflammatory and pro‐regenerative macrophages in skin wounds leading to faster wound closure and re‐epithelialisation as opposed to skin wounds without concomitant TBI. The observed defence response in wounds of combined TBI skin trauma is unexpected and likely contributes to better infection control and survival.

## 2 Materials and Methods

### 2.1 Mouse Model of TBI

TBI was performed using a modified closed weight drop model [7] delivered employing a stereotactic apparatus. Briefly, adult C57BL/6 mice aged p60‐80 were anaesthetised with 5% sevoflurane in 95% O_2_ and treated with 0.1 mg/Kg of buprenorphine prior to the injury. The skin was incised on the midline (2 cm longitudinal incision) to expose the skull, and the mouse was positioned in the apparatus, in which the sides of the head were fixed onto the holding frame. The impact was delivered by dropping a weight of 120 g from a height of 45 cm with a displacement into the skull of 2.5 mm onto specific coordinates of the parietal lobe (*x* = +3.0; *y* = −2.0; *z* = 0.0). 100% of O_2_ was administered after the impact until normal breathing was restored. The skin was sutured with Prolene 6–0 (four sutures) and mice were transferred to their cage with water and food ad libitum. Mice received postoperative analgesia by injection of 0.1 mg/Kg buprenorphine every 8 h until 24 h after impact. Mice were subjected to neurological examination 3 h after TBI using the Neurological Severity Score standardised assessment (performed as previously reported [8, 9]). In agreement with previous reports, all mice had an NSS score less than 1, which defines and corresponds to a mild TBI model. Postoperative assessment and scoring were performed every day until mice were sacrificed. Mice were then euthanised 1 DPI and 7 DPI upon TBI for further experimentation.

### 2.2 RNA Isolation From Skin and Bulk RNA Sequencing

Total RNA was extracted from cranial sutured skin wounds using RNeasy plus kit (Qiagen) according to the manufacturer's instructions. 1–5 μg of extracted total RNA was poly‐A enriched and then subjected to library preparation using Illumina compatible RNA seq libraries. RNA seq libraries were quality controlled through Agilent 5400 Bioanalyzer and by Qubit fluorimeter. Quality validated libraries were sequenced on Illumina NovaSeq × Plus 150 bp paired‐end sequencing system (sequencing depth > 20 million read pair per sample).

The sequencing reads were adaptor trimmed, and quality filtered, followed by aligning to mouse reference genome (mm10) using HISAT2. The aligned reads were used for transcriptome assembly and finally differential expression of genes were measured by DEseq2. Pathway analyses were performed by ClusterProfiler and GO and KEEG pathways. Gene set enrichment analysis was carried out using available R package. Visualisation and representation of the RNA seq data were performed by several custom R scripts in R‐Studio environment.

### 2.3 Quantitative PCR

cDNA was synthesised using 1 μg of total RNA (Maxima first strand cDNA synthesis kit for RT‐qPCR, ThermoFisher Scientific), and 50 ng of synthesised cDNA was employed for quantitative PCR using Power SYBR Green master mix (ThermoFisher Scientific) and QuantStudio 5 Real‐Time PCR System (ThermoFisher Scientific). Log2 fold change of ∆∆Ct (threshold cycle) values was used for analyses. The sequences of the primers used in RT‐qPCR were summarised in Table S1.

### 2.4 Immunostaining

Skin tissues were collected from sutured cranial skin and fixed overnight in 4% PFA in PBS. Subsequently, paraffin embedding was done using an automated tissue processor system (Leica TP1020). Paraffin sections of 5 μm were used for histological analysis. Deparaffinised tissue sections were hydrated through ascending series of ethanol followed by either haematoxylin–eosin staining or immunofluorescence staining. For immunofluorescence staining, the dehydrated sections were used for antigen retrieval and blocking. The blocked sections were then probed with the primary antibodies overnight at 4°C (chicken anti‐cytokeratin 14, ThermoFisher Scientific, Cat: MA5‐11599; rat anti‐CD3, NovousBiologicals, Cat: MAB‐4841; rabbit anti‐filaggrin, BioLegened, Cat: 905804; Rabbit anit‐involucrin, Biolegend, Cat: 924401; Rabbit anti‐collagen III alpha, NovousBiologicals, Cat: NB600‐594; Rabbit anti‐collagen I, NovousBiologicals, Cat: NB600‐408; Rabbit anti‐keratin 10, BioLegened, Cat: 905404; rabbit anti‐loricrin, BioLegend, Cat: 905104; rabbit CD11c, Cell Signalling, Cat: 45581S; mouse anti‐MHC class II, Cell Signalling, Cat: 68258S; rat anti‐F4/80, TheromoFisher Scientific, Cat: 14–4801‐85; anti arginase 1, Cell Signalling, Cat: 93668S). The sections were subsequently rinsed with PBS, followed by an incubation with the fluorophore labelled secondary antibodies (goat anti‐rabbit IgG Alexa Fluor 488, ThermoFisher Scientific, Cat: A11008; goat anti‐rabbit IgG Alexa Fluor 555, ThermoFisher Scientific, Cat: A27039; goat anti‐chicken IgG Alexa Fluor 555, ThermoFisher Scientific, Cat: A21437; goat anti‐rat IgG Alexa Fluor 555, ThermoFisher Scientific, Cat: A21434; goat anti‐mouse IgG Alexa Fluor 555, ThermoFisher Scientific, Cat: A21422). After serial washing with PBS, sections were counter‐stained with DAPI for nuclear staining. Sections were washed once, thereafter mounted in fluorescence mounting media (Dako) and visualised by AxioVision microscope coupled with a CCD camera for image capture. The images were further analysed by ImageJ in terms of fluorescence intensity and area.

### 2.5 ELISA

Mouse sera were explored for insulin growth factor 1 (IGF‐1), S100A8/A9, c‐c motif chemokine 22 (CCL22) and calcitonin gene‐related peptide 1 (CGRP1) measurements using sandwich ELISA‐based, commercially available kits (Mouse/Rat IGF1 ELISA, R&D, Cat: MG100; Mouse S100A8/A9 ELISA, R&D, Cat: DY8596; Mouse CCL22/MDC ELISA, R&D, MCC220; Mouse CGRP1 ELISA, NovousBiologicals, Cat: NBP3‐00522). In brief, diluted serum (500‐fold for IGF1 and 16‐fold for S100A8/A9, CCL22 and CGRP1) was added to the antibody‐coated wells and incubated for 2 h at room temperature, followed by 2 h incubation with the appropriate HRP‐conjugated secondary antibody and finally developed with tetramethylbenzidine (TMB). The exact concentration of target proteins present in the serum was calculated from the given standard curves based on the recorded optical density at 450 nm by a microplate reader (Varioskan LUX Multimodus, ThermoFisher Scientific).

### 2.6 Statistical Analysis

Data analysis was carried out using GraphPad Prism version 9.00. Data are presented as mean ± standard errors of mean (SEM). The significant value was calculated using unpaired two‐tailed Student's *t* test and depicted in graphs as stated in figure legends as follows: *p ≤ 0.05, **p ≤ 0.01, ***p ≤ 0.001, ****p ≤ 0.0001.

## 3 Results

### 3.1 TBI Induces a Protective Transcriptomic Profile in Skin Wounds

**FIGURE 1:**
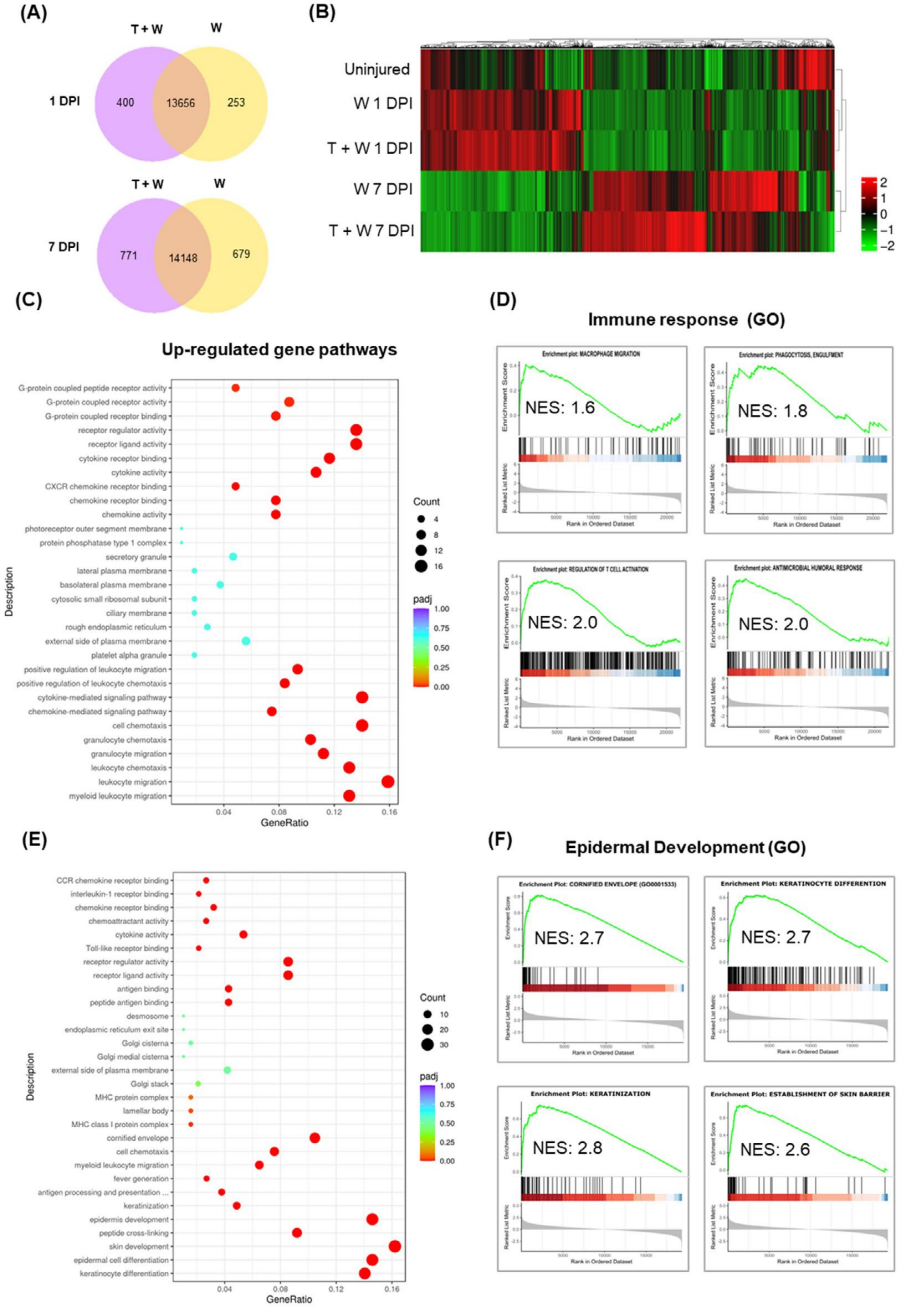
TBI enforces a defensive transcriptomic program accelerating cutaneous wound healing. Transcriptomic analysis was performed in wounded skin at the skull in different mice (*N* = 3) (A) Gene co‐expression diagram comparing number of uniquely expressed genes in the skin wound with and without TBI 1 DPI and 7 DPI. (B) Hierarchical heat map graph of significant differentially regulated genes (DEGs) among all conditions that scale normalised and colour‐coded based on z‐score (C and E) Dot plot of pathway statics of DEGs in Gene Ontology (GO) term in the skin collected from murine incisional wounds 1 and 7 DPI post‐TBI (T + W) compared to wound only (W). Number of enriched genes in each GO term depicted as gene ratio for each corresponding enriched GO term and plotted versus number of counts at each gene set. Each dot represents specific gene set which is colour‐coded based on the *p* adjusted value. Number of counts for each set showed in the dark circles. (D and F) Selected significantly overrepresented gene sets in molecular pathway in T + W condition compared to W only 1 and 7 DPI analysed using Gene Set Enrichment Analysis and calculated based on the normalised enrichment score (NES).

To understand how TBI impacts the healing process of skin wounds, we screened for differential gene expression employing an unbiased RNA seq analysis of bulk skin samples obtained at 1 DPI and 7 DPI from incisonal wounds performed concomitantly with the induction of TBI. Co‐expression analysis showed 400 unique genes that only expressed in TBI with skin wounds compared to 253 uniquely expressed genes in the skin wounds 1 DPI. The number of unique expressed genes in skin wounds with TBI increased to 771 and to 669 after skin wounds 7 DPI (Figure 1A). Moreover, we observed 303 differentially regulated genes (DEGs) including 150 upregulated and 153 downregulated genes in skin collected from TBI mice 1 DPI compared to wound only conditions without preceding TBI (DESeq2, *p* value < 0.05). However, the number of DEGs in TBI versus wound only conditions 7 DPI increased to 555 genes, of which 243 genes upregulated, and 312 genes downregulated. DEGs were clustered in different hierarchical clusters in which differences between transcriptomic profile of TBI skin wound and only skin wounds are globally visible (Figure 1B). Pathway analysis of DEGs genes demonstrate pathways related to innate and adaptive immune response including leukocyte migration and cytokine/chemokine activity in skin wounds collected from mice with TBI 1 DPI versus skin wounds only (Figure 1C). Additional data analysis with Gene set enrichment analysis (GSEA) of GeneOntology GO term showed significant overrepresentation of genes involved in macrophage migration (*Cxcl17*, *Ccl2*), phagocytosis (*Fcer1g*, *Fcgr2b*, *Thbs1*), T cell activation (*Ctla2a*, *Tnfsf18*, *Ceacam1*) antimicrobial humoral response (*Cxcl1*, *Cxcl3*, *Ccl22*) (Figure 1D, Tables [Link], [Link]) and, interestingly, also neuroactive ligand‐receptor interactions (*Calcrl*, *Chrna7*, *Cnr1*, *Gabra1*, *Hrh2*) and sensory perception of pain (*Grin2a*, *Htr2a*, *Cxcr4*) in TBI skin wound 1 DPI (Tables S6 and S7). Using pathway analysis of DEGs of 7 DPI skin wounds identified cornification and keratinization pathways in TBI skin wound (Figure 1E). Further GSEA with GO terms revealed epidermal cornification, keratinocyte differentiation, keratinization and skin barrier formation significantly overrepresented in TBI skin wounds in comparison with wounds only at 7 DPI (Figure 1F). Thus, TBI induces a gene signature indicative of the accelerated regeneration of multilayered, immunologic and epidermal barriers in incisional skin wounds.

### 3.2 TBI Induced an Early Innate and Adaptive Immune Response in Skin Wounds

#### 3.2.1 Boosting of the Innate Macrophage Immune Response After TBI

We investigated further the effect of TBI on cells of innate immunity and their recruitment into the wound site. Genes coding for chemokines involved in macrophage migration to the wound site (*Cxcl3*, *Cxcl17*, *Ccl3*) [10] and genes fostering macrophage phagocytosis and killing (*Il1b*, *Mcoln2*, *Tnfst18* among others) [11, 12] were upregulated.

**FIGURE 2:**
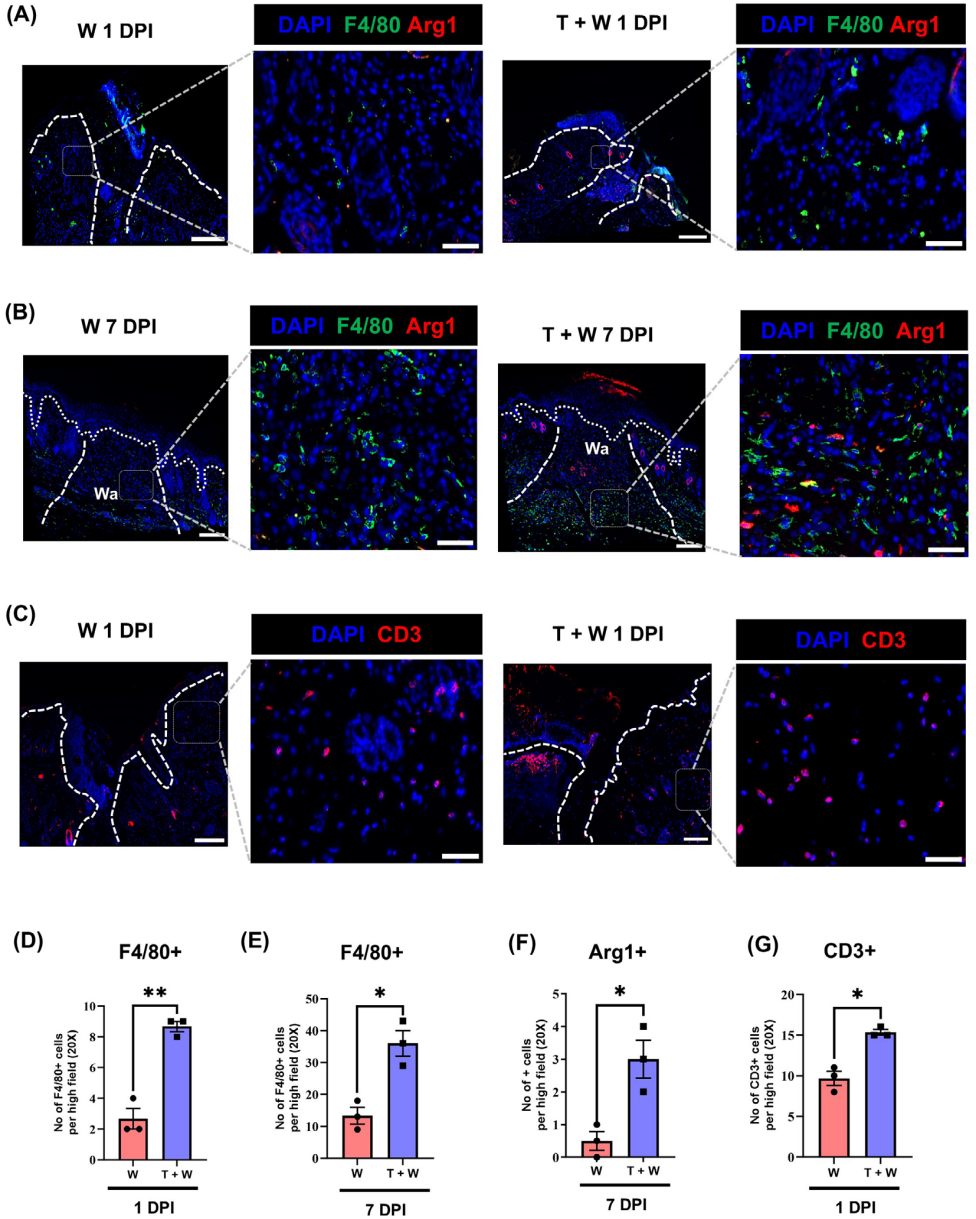
Traumatic brain injury induced early adaptive immune responses and resolution of inflammation in the skin after wounding. (A–C) Immunostaining of wound sections 1 DPI and 7 DPI in skin wound only (W) or post TBI and skin injury (T + W) with antibody against F4/80 as a marker of macrophages in green and Arginase 1 (Arg 1) as marker of pro‐regenerative macrophages as well as CD3 as a marker of T cell in red and nuclear staining in blue. The wound area (Wo) marked with dashed white line. The scale bar sets at 200 and 50 μm, respectively. (D–G) The number of F4/80 positive cells, Arg 1 positive cells and CD3 positive cells in the wound area (Wa) were quantified per high field (20×) and plotted versus the time points post injury. The significant values were calculated using *T* test with Welch's post hoc analysis (*p ≤ 0.05, **p ≤ 0.01, *N* = 3). The scale bar is equivalent to 200 and 50 μm, respectively.

Next, we employed immunostaining on wound sections to further confirm and explore the recruitment of cells involved in innate and adaptive immunity upon TBI. In agreement with bulk‐transcriptomic data, the number of dermal F4/80+ macrophages significantly increased at the wound site in sections from skin wounds from TBI mice versus only skin wounds at 1 DPI (Figure 2A).

We here observed higher numbers of F4/80+ macrophages at 7 DPI; many of them are pro‐regenerative Arginase‐positive macrophages in skin wounds after TBI compared to only skin wounds (Figure 2B,E,F). This is also supported by the gene signature indicative of switching to pro‐regenerative macrophages. Among other genes, the Mdk gene encoding midkine, highly induced in the transcriptome analysis of 7 DPI skin wounds after combined trauma, fosters macrophage polarisation towards pro‐regenerative macrophages [13].

In addition to macrophages, higher numbers of dermal CD11c+, MHCII+ cells were identified in the TBI skin wounds compared to the wound only 7 DPI, which may refer to an increase in the presence of antigen presenting cells (APCs) promoting T cell activation upon TBI in the skin wounds (Figure S1A,B).

#### 3.2.2 Increased Recruitment of T Cells in Skin Wounds After TBI

Along with an increase in macrophages and ACPs, which activate T cells, we observed a significant increase in CD3+ T cells in skin wounds after TBI at 1 DPI when compared to skin wounds only (Figure 2C,G). Interestingly, the expression of the IL‐1β encoding gene *Ilb* is significantly increased in skin wounds after combined trauma as opposed to skin wounds only (see Table S4).

#### 3.2.3 TBI Induced the Expression of Genes Involved in Early Resolution of Inflammation and Antimicrobial Defence Responses

Next, we investigate a set of genes that may contribute to the early resolution of inflammation and its impact on wound healing in the combined‐trauma condition. The genes involved in the resolution of inflammation, which have been obtained from GO pathway analysis, are listed in Table S4. Some of them, like *Ctla2* encoding the cytotoxic T lymphocyte antigen 2a and *Tnfst18* coding for the member 18 of the TNF superfamily, stimulate regulatory T cells and, in consequence, likely suppress effector T cells. *Lilrb4b* encoding the leukocyte Ig‐like receptor 4B downregulates T cell proliferation, antigen presentation, and stimulates CD8+ suppressor cells. Another inflammation‐resolving gene is *Ptpn22*, coding for the protein tyrosine phosphatase N22, which is reported to inhibit antigen receptors and T cell signalling. Finally, *Nfkbid*, coding for the NF‐κB inhibitor delta, inhibits NF‐κB activation and, hence, down‐tones cells of innate immunity and effector functions of inflammatory T cells of the adaptive immunity.

**FIGURE 3:**
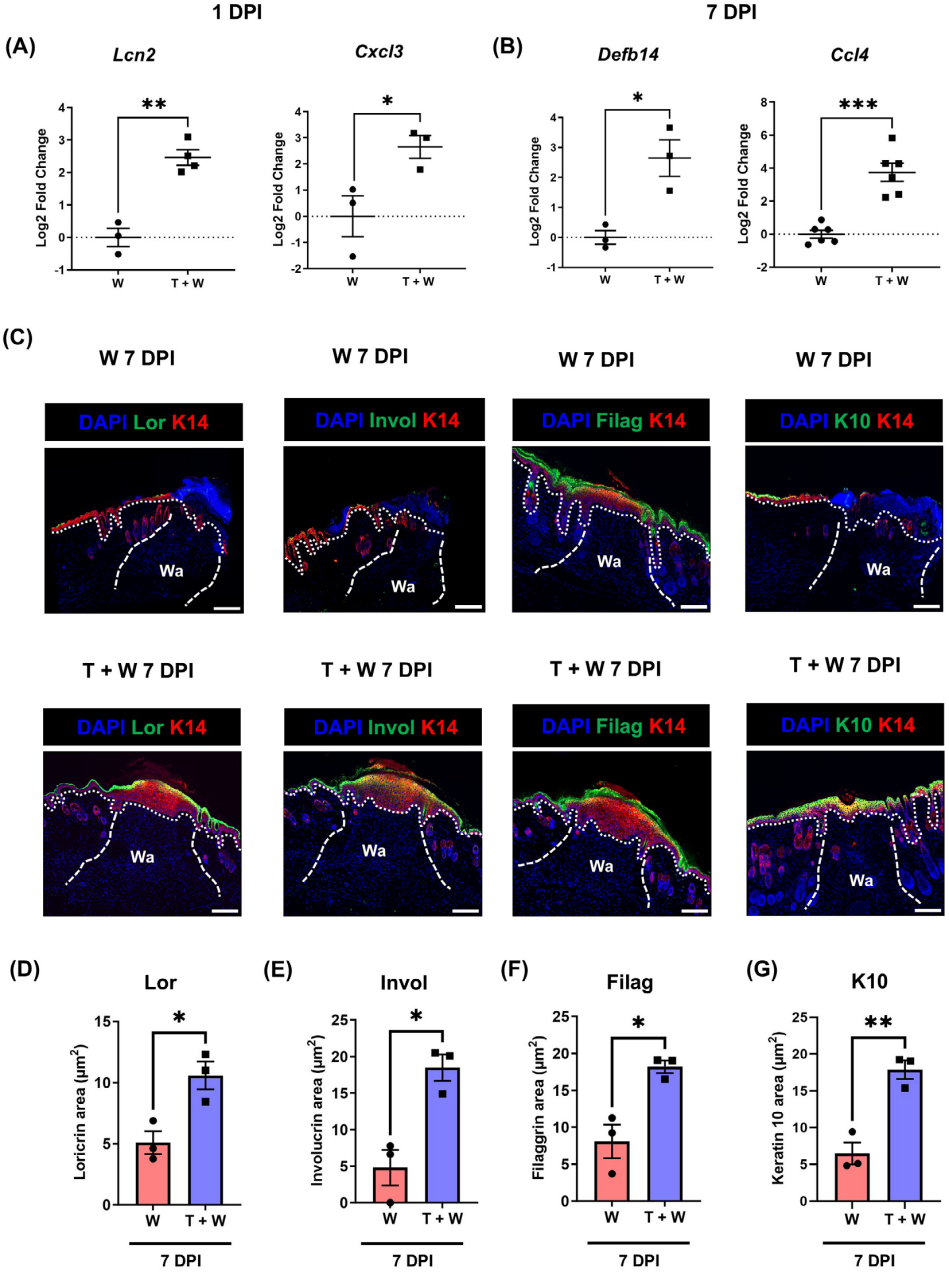
TBI enforces an antimicrobial protective epidermal barrier. (A) Log2 fold change of antimicrobial gene expression, *Lcn2*, *Cxcl3* 1 DPI, and (B) *Defb14*, *Ccl4* 7 DPI in the mouse skin wounds collected after TBI and skin wound (T + W) and post‐skin wound only as quantified by quantitative PCR. (C) Immunostaining of the incision wounds with antibodies against Loricrin (Lor), Involucrin (Invol), Fillagrin (Filag) and Keratin 10 (K10) in green for cornification and keratinization 7 days post‐TBI, nuclear staining in blue and keratin 14 (K14) as marker of undifferentiated epidermis staining in red. Areas (μm^2^) that were positive for (D) Lor, (E) Invol, (F) Filag, and (G) K10 were quantified using area calculator function in imageJ and plotted against conditions using GraphPad Prism. The significant values were calculated using unpaired *T*‐test with Welch's post hoc analysis (*p ≤ 0.05, **p ≤ 0.01, ***p ≤ 0.001, *N* = 3). The scale bar is equivalent to 200 μm.

Furthermore, we explored antimicrobial peptides by RNA seq and by qPCR. Antimicrobial humoral response was overrepresented in TBI 1 DPI skin wounds (Figure 1D and Table S5). A significant up‐regulation of *Lcn2* coding for lipocalin‐2 was detected in TBI skin wounds 1 DPI (Figure 3A), while antimicrobial genes like *Defb14* encoding defensin beta 14 and *Ccl4* were upregulated 7 DPI in skin wounds after combined trauma (Figure 3B).

### 3.3 TBI Promoted Expression of Genes and Proteins Associated With Cornification and Keratinization of Skin Wounds

A significant enrichment of cornification and keratinization pathways was depicted in global gene expression data in Figure 1E,F, which was additionally confirmed for *loricrin* and *hornerin* by qPCR (Figure S2E,F). To further evaluate the formation of the epidermal cornified envelope and re‐epithelialization upon TBI, wound sections were stained with antibodies against molecules of the epidermal cornification and keratinization, including loricrin, involucrin, filaggrin, and Keratin 10 (K10). Here we observed a significant increase in the expression of these proteins, as indicated by an extended, more intensely stained area μm^2^ for all the positively stained cornification molecules after 7 DPI TBI compared to the skin wounds only (Figure 3C–G). This observation suggests a profoundly reinforced formation of the epidermal barrier upon TBI. In addition, we noticed a remarkably thicker epidermis in the TBI wound sections, as indicated by a thick layer stained with Keratin 14 (K14), the proliferation‐associated keratin, further protecting the dermis and hypodermis tissues. Collectively, these data suggest that combined TBI and skin trauma may accelerate the formation of an effective epidermal barrier.

### 3.4 TBI Induced Dermal Collagen Deposition and Myofibroblast Transition in Incisional Skin Wounds

**FIGURE 4:**
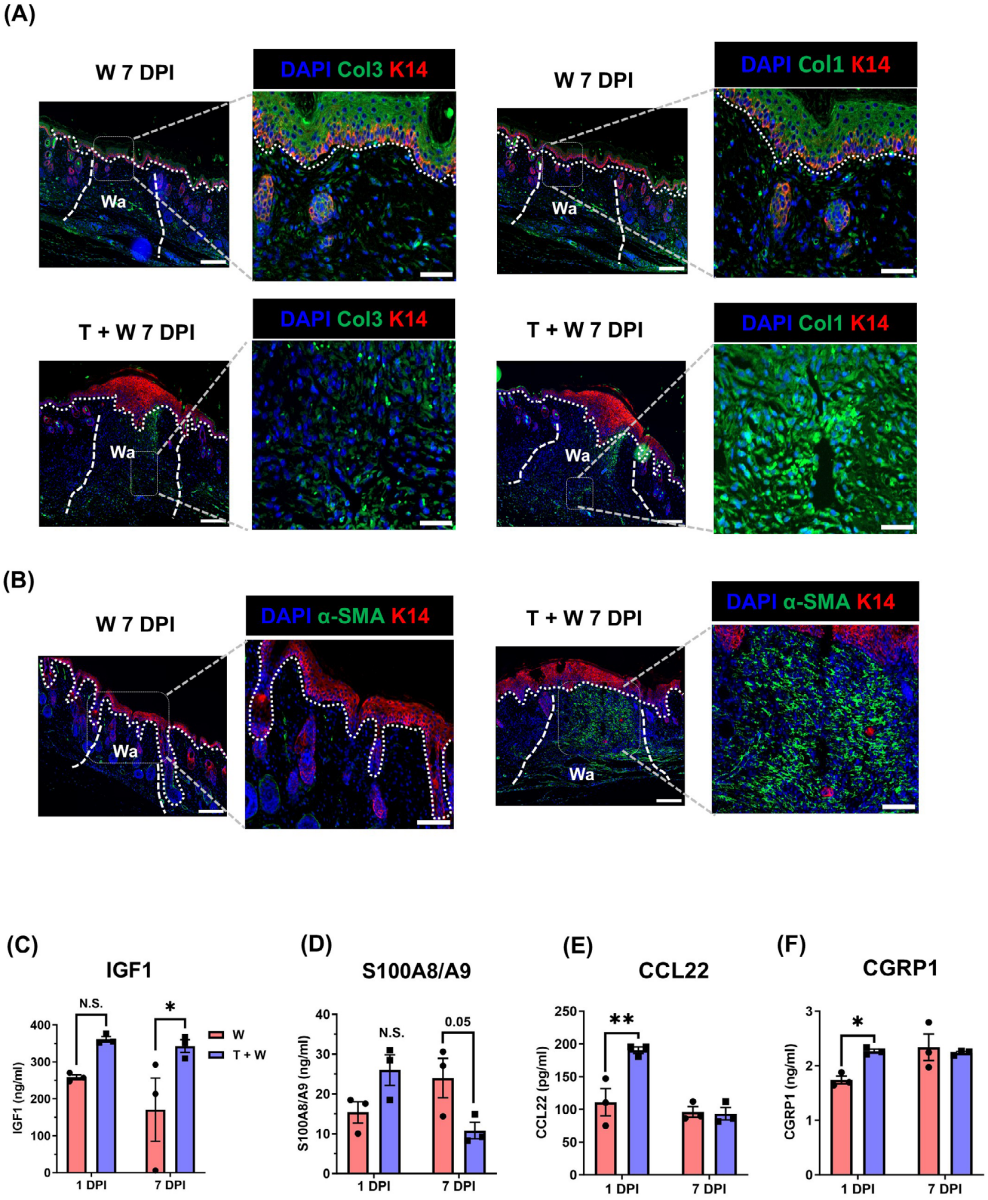
TBI enhances a protective dermal barrier and releases IGF‐1 and CCL22 enhancing wound healing. (A) Immunostaining of wound sections from skin wound only (W) and TBI with skin wounds (T + W) 7 DPI stained with antibodies against collagen III (Col 3) and collagen I (Col 1) in green colour, with Keratin14 (K14) in red and DAPI as a nuclear marker 7 DPI. (B) Wound sections stained against alpha smooth muscle Actin (αSMA) in green, K14 in red and nuclear staining in blue. Wound area is marked using white dashed line (Wo) (C–F) Serum collected from mice 1 DPI and 7 DPI with and without TBI (T + W, W) subjected to commercial ELISA for screening pro‐wound healing parameters including IGF1, S100A8/A9, CCL22 and CGRP1. Corresponding concentrations were calculated using curve fit function and were plotted versus the time points. Significant values were calculated using two‐way ANOVA with Sidak's post hoc correction (*p ≤ 0.05, **p ≤ 0.01, *N* = 3). The scale bar is equivalent to 200 and 50 μm, respectively.

As closure of the wounds with collagen accumulation and myofibroblast is essential for a functional dermal barrier, we determined the levels of collagen deposition and α‐SMA+ myofibroblasts employing immunostaining of sections of skin wounds from TBI and only skin wounds at 7 DPI. We demonstrate that collagen type I replaces collagen type III more rapidly and to a higher extent in the TBI skin wounds, whereas in the only skin wounds exclusively collagen type III is only a minor extent visible in the wound area without any collagen type I deposition even at 7 DPI (Figure 4A). These data highlight the profound acceleration of collagen deposition and maturation in skin wound after combined trauma as opposed to skin wounds only. Moreover, we observed accumulation of α‐SMA‐expressing cells in the wound incision cut area in the TBI skin wounds, which was not observed in the control condition (Figure 4B). These observations suggest an accelerated maturation phase characterised by the formation of an effective dermal barrier and, consequently, potentially faster wound healing following TBI.

### 3.5 TBI Released Systemic Factors Into the Serum May Enhance Skin Wound Healing

To understand how after TBI systemic brain responses may contribute to the observed acceleration of cutaneous wound healing, we evaluated the concentrations of distinct wound healing promoting factors, like IGF1, S100A8/A9, CCL22, and CGRP1 in the serum after TBI. Here we demonstrate that after TBI, IGF1 concentrations significantly increased in the serum of mice 7 DPI after TBI (Figure 4C). Moreover, S100A8/A9 serum concentrations increased non‐significantly 1 DPI and reduced 7 DPI (Figure 4D). In addition, we found concentrations of CCL22 or macrophage‐derived chemokine to be significantly elevated upon TBI at 1 DPI (Figure 4E). Interestingly, we found a profound increase of CGRP1 in the serum of mice with combined TBI skin trauma as opposed to mice subjected to skin trauma only. The increase occurred at 1 DPI, while later it diminished (Figure 4F).

## 4 Discussion

The major finding of this study is that a mild brain trauma when concomitantly occurring with skin injury results in faster healing of the skin wound. Surprisingly, different phases of wound healing are boosted after combined TBI—skin trauma with a more rapid formation of an immune barrier, an epidermal barrier, and a dermal barrier, collectively likely counteracting infection. The release of systemic factors in the circulation after TBI, like IGF‐1, CCL22, a macrophage chemotactic protein, and the calcitonin‐related peptide CGRP1 are likely involved in the acceleration of the sequence of distinct wound phases after skin injury.

We here found an early increase in the recruitment of pro‐inflammatory macrophages followed by an early switch to pro‐healing, regenerative macrophages in skin wounds after TBI as opposed to skin wounds only. In line with our findings, upregulation of macrophage pro‐regenerative genes was reported in meningeal tissues of mice undergoing mild TBI 7 DPI [14]. Macrophages are indispensable for wound healing as their phagocytotic activity cleans the wound site from tissue debris and invading microorganisms during the inflammation phase. In addition, by their enhanced paracrine crosstalk, macrophages activate other cells critical for proliferation, angiogenesis, and collagen deposition. The inability to recruit or switch from pro‐inflammatory macrophages to pro‐regenerative macrophages is causal for the non‐healing state of chronic venous leg ulcers [15]. Wound transplantation of macrophages accelerated wound healing in vivo [16], while depletion of macrophages led to delayed and inefficient wound healing [17, 18]. In addition to the better recruitment and switch from pro‐inflammatory to regenerative macrophages, we observed higher numbers of CD11c + cells in skin wounds after TBI, a result which correlates with the here found protective gene signature in skin wounds upon TBI. The protective gene signature includes significantly up‐regulated genes encoding for *Il1b*, *Cd80*, and *Cd209a*, which are crucial for antigen presentation in skin wounds after combined trauma (Table S4). IL‐1 has the capacity to enforce maturation of antigen presenting cells (APC) [19] with upregulation of co‐stimulatory molecules and distinct T cell activating cytokines. In contrast to our data, a recent study [20] shows that the number of CD11c + APCs are reduced in the intact skin collected from the mouse ear after moderate 7 DPI TBI. This discrepancy is likely due to the difference in APC numbers between intact skin after TBI and skin injury after TBI, or possibly to the skin collection site. APCs contributed to accelerated wound healing in a burn injury mouse model [21]. CD11c is a known marker of murine APCs and its depletion was shown to delay wound healing in mice [22]. In addition, higher numbers of T cells were observed 1 DPI in combined skin wound and TBI. While we did not focus on exploring different T cell populations in our study, increased numbers of regulatory T cells (Tregs) were reported in the peripheral blood of TBI patients 1 day after TBI [23]. Interestingly, local administration of Tregs improves wound healing of the skin by promoting the anti‐inflammatory properties of macrophages [24]. Our observations indicate a pro‐inflammatory response in the skin wound after combined trauma as observed by increased numbers of pro‐inflammatory macrophages, followed by a strong, faster anti‐inflammatory response mediated by Arg + F4/80+ macrophages; the anti‐inflammatory role of enhanced numbers of T cells is likely and awaits further specification in future studies. One limitation of this study is that we did not dissect which T cell subpopulation increased in number and what it means for their functional activity. Nevertheless, the benefit of an accelerated resolution of inflammation by pro‐regenerative macrophages without any doubt leads to the protection of normal tissue from profound collateral damage of an extended inflammation as observed in skin wounds without TBI. If the damage is controlled, re‐epithelialisation, wound contraction, and collagen deposition in the maturation phase could be earlier accomplished, thus effectively and faster sealing the wound against microbial invasion.

As there is still some threat of microbial invasion in late phases of wound healing, we specifically explored the impact of antimicrobial peptides as efficient effector molecules of innate immune cells and of the epidermal barrier. In this study, we demonstrated upregulated antimicrobial gene expression of *Cxcl1* 1 DPI and *Lcn2*, *Def14b*, and *Ccl4* in late time point combined skin wounds and TBI. Cxcl1 is a keratinocyte‐derived chemokine that promotes recruitment of leukocytes and stimulates sensory neurones via CXCR2 signalling [25]. CXCR2 deleted mice, in fact, showed delayed wound healing, which indicates a supportive role of CXCR2 signalling in wound healing [26]. LCN2, DEFB14, and CCL4 play a role in antimicrobial defence preventing bacterial growth [27, 28, 29]. These data highlight a persisting and yet time‐dependent elevation in antimicrobial gene expression in the skin wounds upon TBI, which, in addition to contributing to the early inflammatory response, may contribute a defensive line against pathogens, reinforcing and facilitating re‐epithelialisation (Figure S2). In line with our data, contusional TBI was reported to induce an antimicrobial gene signature in the mouse brain with an upregulation in *Lcn2*, *S100a8* and *Ccl4* [29].

As the cornified envelope of the differentiated epidermis provides a strong epidermal barrier against environmental threats, we had been interested in exploring its reconstitution after combined TBI and skin injury. The cornified envelope of the skin is the outermost layer and forms a highly insoluble structure beneath the cell membrane during terminal differentiation of epidermal cells [30], and it protects the skin and the organism from microbial and allergen invasion or chemical damage, counteracts fluid loss and provides the skin with stability and flexibility to resist mechanical forces. These properties of the cornified envelope are due to at least 10 proteins, among them loricrin, filaggrin, involucrin, hornerin, and K10, which are crosslinked with each other by epidermal transglutaminases. In this study, we found not only an upregulated cornification gene signature, but also enhanced and earlier occurring expression of certain cornification proteins like loricrin, filaggrin, involucrin, and K10. These data highlight elevated epidermal barrier gene and protein expression, which might suggest enhanced barrier function and recovery of the cornified envelope of the skin wounds upon TBI.

The dermal barrier occurs in the maturation phase of wound healing where collagen type III is gradually substituted by collagen type I, and certain subsets of fibroblasts differentiate into α‐smooth muscle cells (α‐SMA)‐expressing myofibroblasts, which are responsible for collagen synthesis and wound contraction [31]. Myofibroblast‐driven wound contraction contributes to wound closure and thereby counteracts infection, while the switch from collagen type III to collagen type I enhances the tensile strength of the wound and, in consequence, protects from rupture of the newly formed restoration tissue. We here identified higher deposition of collagen type I as well as increased staining for α‐SMA in combined skin wound and TBI, suggesting improved wound closure and stability.

As to the questions of how TBI may contribute to better healing of different phases of concomitantly occurring skin wounds, we searched for likely candidates. We identified higher serum levels of IGF1, S100A8/A9, and CCL22 at 1 DPI in mice subjected to combined skin wounds and TBI. Previously and unpublished, we identified that the IGF1 concentration in the serum was elevated 24 h after blunt chest trauma in vivo. IGF1 is a known growth factor that not only supports neurogenesis in the brain after TBI [32], but also contributes to the resolution of inflammation, vascularisation, re‐epithelialisation, and collagen deposition in acute wounds [33, 34, 35]. Additionally, S100A8/A9, a neutrophil‐derived danger‐associated molecular pattern molecule, facilitates recruitment of leukocytes upon injury and enhances healing of wounds injected with S100A8/A9‐primed mesenchymal stromal cells [36]. The observed early increase in serum levels of macrophage‐derived CCL22 was in line with an increased number of macrophages in skin wounds. An enhanced number of macrophages in the human brain was also reported 1 day after TBI [37]. CCL22 is involved in APC‐mediated pain sensation in incisional skin wounds [38] and regulates T cell functions [39]. Even though we uncovered these soluble factors and data are published on how they may impact wound healing, currently we did not present functional proof.

Based on our results from unbiased transcriptome analysis, we identified the enrichment of genes in the neuroactive ligand‐receptor interactions and sensory perception of pain. This, in conjunction with the known impact of neuroendocrine substances on the skin [40], led us to assess the most reasonable candidate CGRP, a 37‐amino‐acid neuropeptide which is mandatory for cutaneous wound healing, as wound healing is delayed in CGRP knockout mice [41]. It is remarkable that CGRP has been described to attenuate inflammation [42], to induce IGF‐1 [43] and thus may qualify as a major regulator of TBI‐induced acceleration of wound healing and infection protection. However, mechanistic proof needs to be addressed in future experiments. These data highlight and suggest that the positive impact of TBI on wound healing of the skin may be mediated through a paracrine effect of circulatory factors on the skin wound microenvironment.

Though the major players have not been identified, previous studies have shown that TBI improves osteogenesis and stimulates bone healing in different bone fracture models [44, 45, 46] through stimulation of sympathetic nerve or neuronal‐derived extracellular vesicles containing bone supporting molecules including microRNAs. Interestingly, in these models an augmented inflammatory response contributed to the accelerated bone fracture healing by enhancing callus formation [45]. These data, when compared to our data, highlight that the interorgan interactions between brain and peripheral organs are not alike, but rather depend on the individual injured organ.

In conclusion, we here provide correlative evidence that TBI induces a multilayered protective response of the skin overall leading to rapid and improved cutaneous wound healing. In future, it remains to be elucidated whether soluble factors released after TBI such as IGF1, CCL22, and CGRP1 enforce a systemic effect that leads to a protective response in the skin.

## Conflicts of Interest

The authors declare no conflicts of interest.

## Supporting information


**Figure S1:** TBI induced early resolution of inflammation and long‐lasting innate immunity in the skin after wounding. (A) Immunostaining of wound sections 7 DPI in skin wound only (W) or post TBI and skin injury (T + W) with antibody against major histocompatibility complex molecules class II (MHCII) in red and CD11c in green as marker of antigen presenting cells and nuclear staining in blue. The wound area (Wo) marked with dashed white line. The scale bar sets at 200 and 50 μm, respectively. (B) The number of CD11c/MHCII positive cells in the wound area (Wa) was quantified per high field (20×) and plotted versus the time point post injury. The significant values were calculated using two‐way ANOVA with Sidak's multiple comparison analysis (**p ≤ 0.01, ***p ≤ 0.001, ****p ≤ 0.0001 *N* = 3). The scale bar is equivalent to 200 and 50 μm, respectively.

**Figure S2:** TBI enforces a skin barrier protective gene expression profile. (A–D) Log2 fold change of immune response gene expression (*Cxcl1*, *Ccl3*, *Il1a*, *Il1b*) 1 DPI, (E–H) cornification and keratinization genes (*Hrnr*, *Lor*, *Krt23*, *Krt2*) 7 DPI, and (I–L) innate immunity genes (*S100a8*, *S100a9*, *Il1a*, *Gabarp*) 7 DPI in the mouse skin wounds collected after TBI and skin wound (T + W) and post‐skin wound only as quantified by quantitative PCR. The significant values were calculated using *T*‐test with Welch's post hoc analysis (*p ≤ 0.05, **p ≤ 0.01, ***p ≤ 0.001, ****p ≤ 0.0001).

**Table S1:** List of used primers for RT‐PCR in this study.

**Table S2:** Significantly core enriched genes in mouse skin wounds 1 day post traumatic brain injury as assessed by gene set enrichment analysis of macrophage migration Gene Ontology term.

**Table S3:** Overrepresented core enriched genes in mouse skin wounds 1 day post traumatic brain injury as assessed by gene set enrichment analysis of phagocytosis Gene Ontology term.

**Table S4:** Overrepresented core enriched genes in mouse skin wounds 1 day post traumatic brain injury as assessed by gene set enrichment analysis of T cell activation Gene Ontology term.

**Table S5:** Overrepresented core enriched genes in mouse skin wounds 1 day post traumatic brain injury as assessed by gene set enrichment analysis of antimicrobial humoral response Gene Ontology term.

**Table S6:** Overrepresented core enriched genes in mouse skin wounds 1 day post traumatic brain injury as assessed by gene set enrichment analysis of neuroactive ligand‐receptor interactions Gene Ontology term.

**Table S7:** Overrepresented core enriched genes in mouse skin wounds 1 day post traumatic brain injury as assessed by gene set enrichment analysis of sensory perception of pain Gene Ontology term.

## Data Availability

The datasets in this study are available in the following database: RNA seq data: Gene expression. Omnibus GSE288594 (https://www.ncbi.nlm.nih.gov/geo/query/acc.cgi?acc=GSE288594).
