# Supplementary figures and images for: Traumatic Brain Injury Induces Early Barrier Protective Responses in Incisional Skin Wounds Accelerating Cutaneous Wound Healing
Source: Wound Repair Regen. 2025 Aug 29;33(5):e70079. doi: 10.1111/wrr.70079 (PMC12395893; doi:10.1111/wrr.70079)

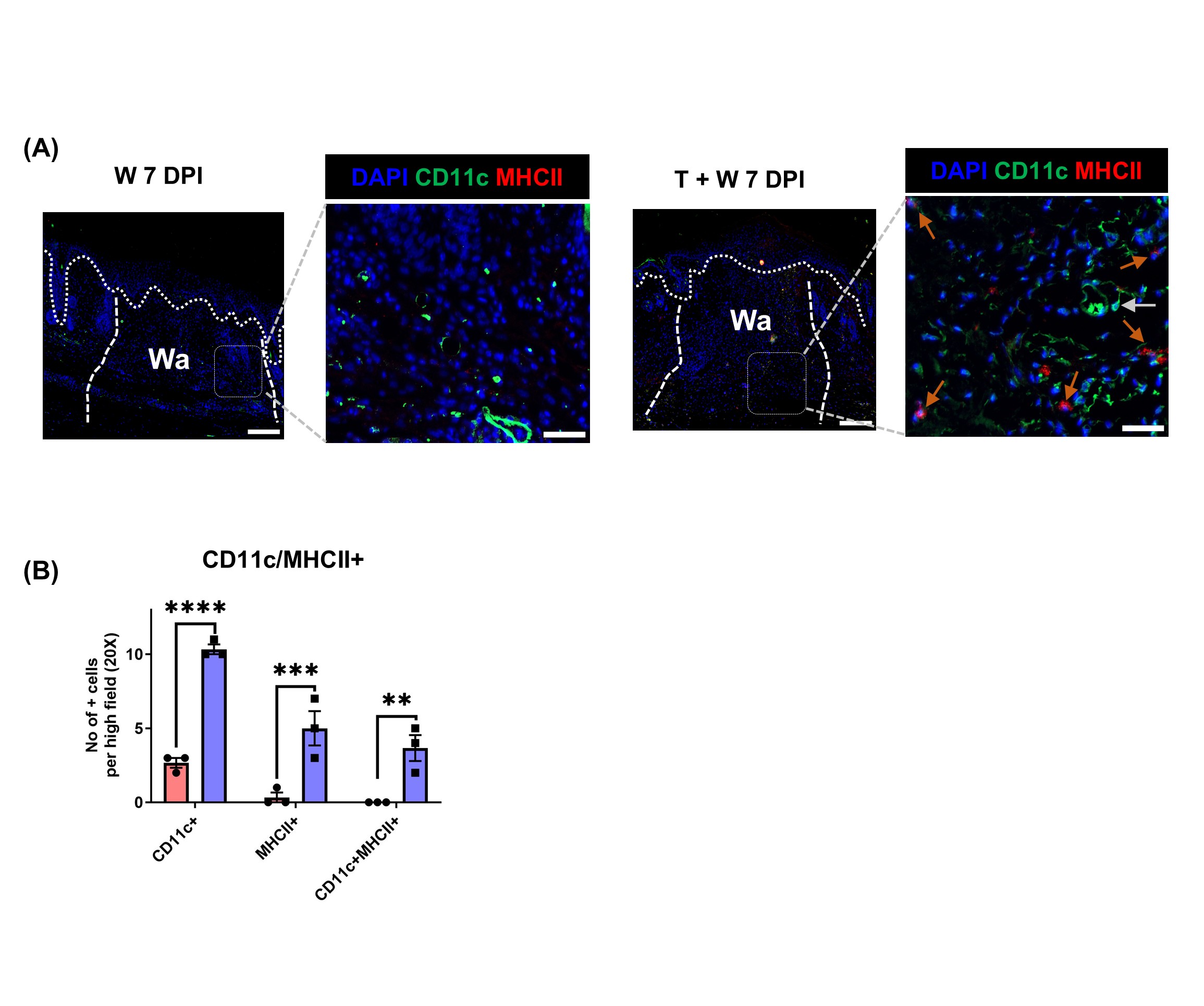

Supplement: Supplementary file 1 — Figure S1: TBI induced early resolution of inflammation and long‐lasting innate immunity in the skin after wounding. (A) Immunostaining of wound sections 7 DPI in skin wound only (W) or post TBI and skin injury (T + W) with antibody against major histocompatibility complex molecules class II (MHCII) in red and CD11c in green as marker of antigen presenting cells and nuclear staining in blue. The wound area (Wo) marked with dashed white line. The scale bar sets at 200 and 50 μm, respectively. (B) The number of CD11c/MHCII positive cells in the wound area (Wa) was quantified per high field (20×) and plotted versus the time point post injury. The significant values were calculated using two‐way ANOVA with Sidak's multiple comparison analysis (**p ≤ 0.01, ***p ≤ 0.001, ****p ≤ 0.0001 N = 3). The scale bar is equivalent to 200 and 50 μm, respectively. [file WRR-33-0-s007.jpg]

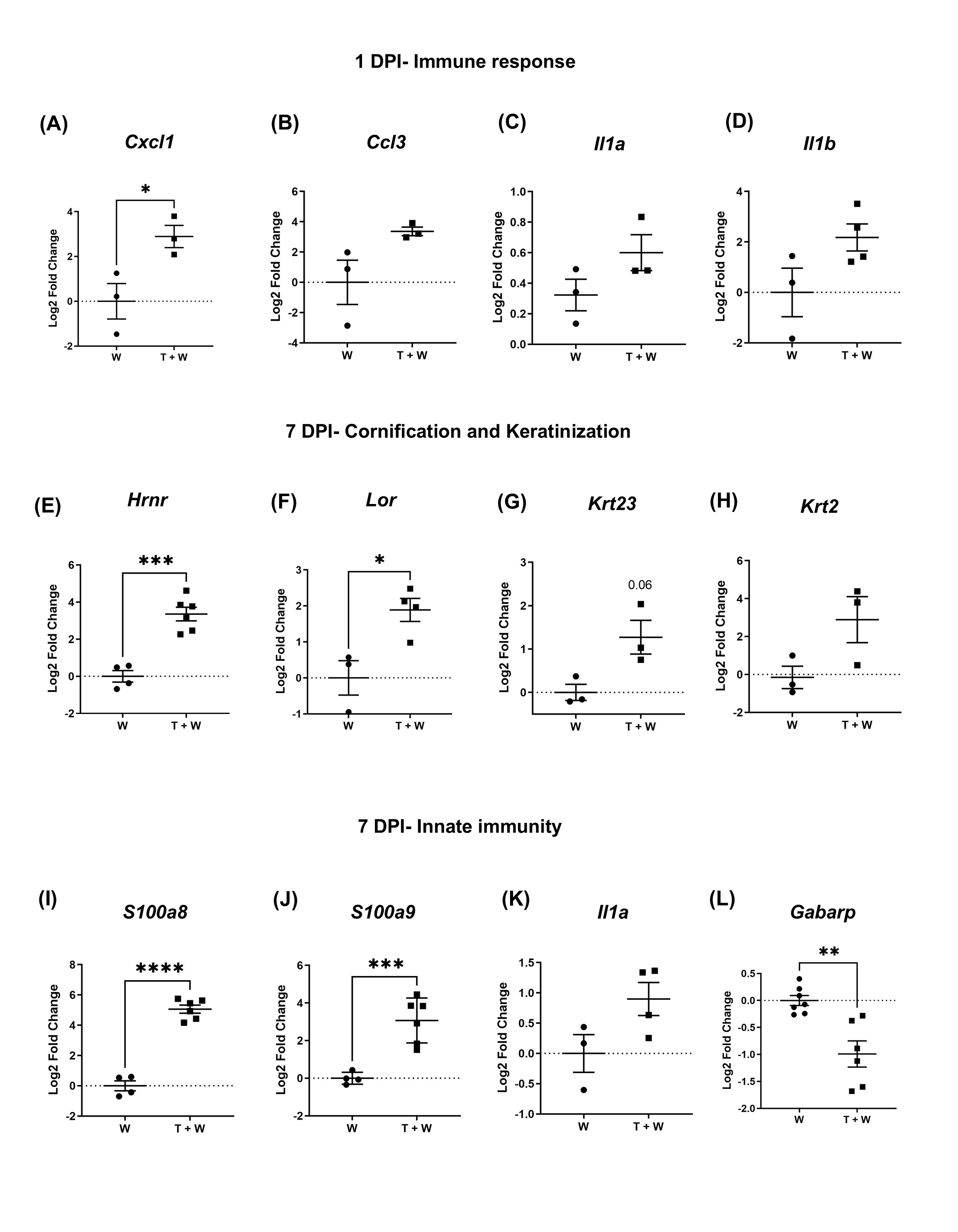

Supplement: Supplementary file 2 — Figure S2: TBI enforces a skin barrier protective gene expression profile. (A–D) Log2 fold change of immune response gene expression (Cxcl1, Ccl3, Il1a, Il1b) 1 DPI, (E–H) cornification and keratinization genes (Hrnr, Lor, Krt23, Krt2) 7 DPI, and (I–L) innate immunity genes (S100a8, S100a9, Il1a, Gabarp) 7 DPI in the mouse skin wounds collected after TBI and skin wound (T + W) and post‐skin wound only as quantified by quantitative PCR. The significant values were calculated using T‐test with Welch's post hoc analysis (*p ≤ 0.05, **p ≤ 0.01, ***p ≤ 0.001, ****p ≤ 0.0001). [file WRR-33-0-s006.jpg]
