# Supplementary material for: Traumatic Brain Injury Induces Early Barrier Protective Responses in Incisional Skin Wounds Accelerating Cutaneous Wound Healing
Source: Wound Repair Regen. 2025 Aug 29;33(5):e70079. doi: 10.1111/wrr.70079 (PMC12395893; doi:10.1111/wrr.70079)
Supplement: Supplementary file 3 — Table S1: List of used primers for RT‐PCR in this study. [file WRR-33-0-s002.docx]

| **Number** | **Gene** | **Primer sequence (5' - 3')** | **RefSeq number** |
| --- | --- | --- | --- |
| **1** | *Lcn2* | F - CTACAATGTCACCTCCATCCTG | NM_008491 |
|  |  | R - CCTGTGCATATTTCCCAGAGT |  |
| **2** | *Cxcl1* | F - CCAAACCGAAGTCATAGCCA | NM_008176 |
|  |  | R - GTGCCATCAGAGCAGTCT |  |
| **3** | *Cxcl3* | F - CAGAAGTCATAGCCACTCTCAAG | NM_203320 |
|  |  | R - GCTCAGCTGGACTTGCC |  |
| **4** | *Defb14* | F - CTCATCTTGTTCTTGGTGCCT | NM_183026 |
|  |  | R - ACATCGACCTATTTGTTCTTCCT |  |
| **5** | *S100a8* | F - ATGACTTCAAGAAAATGGTCACTAC | NM_013650 |
|  |  | R - CCACACCCACTTTTATCACCA |  |
| **6** | *S100a9* | F - GGAATTCAGACAAATGGTGGAAG | NM_009114 |
|  |  | R - CATCAGCATCATACACTCCTCA |  |
| **7** | *Ccl4* | F - CTCTCTCTCCTCTTGCTCGT | NM_013652 |
|  |  | R - GTCTCATAGTAATCCATCACAAAGC |  |
| **8** | *Lor* | F - CACATCAGCATCACCTCCTTC | NM_008508 |
|  |  | R - TCTTTCCACAACCCACAGG |  |
| **9** | *Hrnr* | F - AAGCAACATCAGTCTCCATCC | NM_133698 |
|  |  | R - TGTCACAGTTCCCATATTCAGTG |  |
| **10** | *Actb* | F - GATTACTGCTCTGGCTCCTAG | NM_007393 |
|  |  | R - GACTCATCGTACTCCTGCTTG |  |

**Table S1:** List of used primers for RT-PCR in this study.
