# Supplementary material for: Traumatic Brain Injury Induces Early Barrier Protective Responses in Incisional Skin Wounds Accelerating Cutaneous Wound Healing
Source: Wound Repair Regen. 2025 Aug 29;33(5):e70079. doi: 10.1111/wrr.70079 (PMC12395893; doi:10.1111/wrr.70079)
Supplement: Supplementary file 4 — Table S2: Significantly core enriched genes in mouse skin wounds 1 day post traumatic brain injury as assessed by gene set enrichment analysis of macrophage migration Gene Ontology term. [file WRR-33-0-s004.docx]

| **Entrez_id** | **Gene_name** | **Base mean** | **Log2 fold change** | **lfcSE** | **Stat** | **p value** |
| --- | --- | --- | --- | --- | --- | --- |
| 232983 | **Cxcl17** | 4.382 | 4.557 | 1.636 | 2.786 | 0.005 |
| 240873 | **Tnfsf18** | 38.417 | 1.217 | 0.484 | 2.514 | 0.012 |
| 20302 | **Ccl3** | 452.749 | 0.891 | 0.409 | 2.179 | 0.029 |
| 68279 | **Mcoln2** | 1149.632 | 0.695 | 0.330 | 2.105 | 0.035 |
| 17242 | **Mdk** | 59.511 | 1.032 | 0.525 | 1.966 | 0.049 |
| 20296 | Ccl2 | 1116.434 | 0.762 | 0.441 | 1.729 | 0.084 |
| 21825 | Thbs1 | 11786.363 | 0.685 | 0.400 | 1.712 | 0.087 |
| 68050 | Akirin1 | 1481.917 | 0.379 | 0.255 | 1.486 | 0.137 |
| 13615 | Edn2 | 8.006 | 1.097 | 0.812 | 1.350 | 0.177 |
| 12273 | C5ar1 | 1427.803 | 0.466 | 0.347 | 1.345 | 0.179 |
| 64095 | Gpr35 | 755.401 | 0.483 | 0.371 | 1.303 | 0.193 |
| 57781 | Cd200r1 | 366.729 | 0.476 | 0.383 | 1.243 | 0.214 |
| 56792 | Stap1 | 88.475 | 0.427 | 0.352 | 1.213 | 0.225 |
| 58217 | Trem1 | 680.923 | 0.614 | 0.511 | 1.201 | 0.230 |

**Table S2:** Significantly core enriched genes in mouse skin wounds 1 day post traumatic brain injury as assessed by gene set enrichment analysis of macrophage migration Gene Ontology term.
