# Supplementary material for: Traumatic Brain Injury Induces Early Barrier Protective Responses in Incisional Skin Wounds Accelerating Cutaneous Wound Healing
Source: Wound Repair Regen. 2025 Aug 29;33(5):e70079. doi: 10.1111/wrr.70079 (PMC12395893; doi:10.1111/wrr.70079)
Supplement: Supplementary file 5 — Table S3: Overrepresented core enriched genes in mouse skin wounds 1 day post traumatic brain injury as assessed by gene set enrichment analysis of phagocytosis Gene Ontology term. [file WRR-33-0-s008.docx]

| **Entrez_id** | **Gene_name** | **Base mean** | **Log2 fold change** | **lfcSE** | **Stat** | **p value** |
| --- | --- | --- | --- | --- | --- | --- |
| 14130 | **Fcgr2b** | 3423.298 | 0.758 | 0.280 | 2.708 | 0.007 |
| 14127 | **Fcer1g** | 1306.500 | 0.810 | 0.365 | 2.217 | 0.027 |
| 14131 | Fcgr3 | 1627.843 | 0.630 | 0.346 | 1.821 | 0.069 |
| 21825 | Thbs1 | 11786.363 | 0.685 | 0.400 | 1.712 | 0.087 |
| 56644 | Clec7a | 861.786 | 0.692 | 0.420 | 1.648 | 0.099 |
| 224840 | Treml4 | 71.238 | 0.688 | 0.448 | 1.536 | 0.125 |
| 11687 | Alox15 | 102.918 | 0.620 | 0.424 | 1.462 | 0.144 |
| 16016 | Ighg2b | 3.272 | 1.694 | 1.331 | 1.273 | 0.203 |
| 56792 | Stap1 | 88.475 | 0.427 | 0.352 | 1.213 | 0.225 |
| 20288 | Msr1 | 1404.999 | 0.524 | 0.452 | 1.159 | 0.246 |
| 12491 | Cd36 | 6160.504 | 0.451 | 0.409 | 1.102 | 0.270 |
| 228787 | Xkr7 | 1.021 | 2.182 | 2.259 | 0.966 | 0.334 |
| 70676 | Gulp1 | 371.571 | 0.381 | 0.404 | 0.943 | 0.346 |
| 347708 | Dppa1 | 1.407 | 1.776 | 1.894 | 0.938 | 0.348 |
| 12540 | Cdc42 | 12134.544 | 0.180 | 0.255 | 0.707 | 0.480 |
| 12725 | Clcn3 | 1748.028 | 0.245 | 0.359 | 0.683 | 0.494 |
| 19016 | Pparg | 799.970 | 0.166 | 0.243 | 0.683 | 0.494 |
| 14063 | F2rl1 | 1372.518 | 0.165 | 0.258 | 0.639 | 0.523 |
| 56208 | Becn1 | 1890.173 | 0.151 | 0.238 | 0.636 | 0.525 |
| 83382 | Siglece | 283.618 | 0.296 | 0.482 | 0.615 | 0.539 |
| 140580 | Elmo1 | 631.894 | 0.182 | 0.304 | 0.600 | 0.549 |
| 171283 | Havcr1 | 11.464 | 0.514 | 0.888 | 0.579 | 0.563 |
| 216190 | Appl2 | 2162.589 | 0.159 | 0.278 | 0.573 | 0.566 |

**Table S3:** Overrepresented core enriched genes in mouse skin wounds 1 day post traumatic brain injury as assessed by gene set enrichment analysis of phagocytosis Gene Ontology term.
