# Supplementary material for: Traumatic Brain Injury Induces Early Barrier Protective Responses in Incisional Skin Wounds Accelerating Cutaneous Wound Healing
Source: Wound Repair Regen. 2025 Aug 29;33(5):e70079. doi: 10.1111/wrr.70079 (PMC12395893; doi:10.1111/wrr.70079)
Supplement: Supplementary file 6 — Table S4: Overrepresented core enriched genes in mouse skin wounds 1 day post traumatic brain injury as assessed by gene set enrichment analysis of T cell activation Gene Ontology term. [file WRR-33-0-s005.docx]

| **Entrez_id** | **Gene_name** | **Base mean** | **Log2 fold change** | **lfcSE** | **Stat** | **p value** |
| --- | --- | --- | --- | --- | --- | --- |
| 13024 | **Ctla2a** | 961.125 | 0.912 | 0.267 | 3.420 | 0.001 |
| 26365 | **Ceacam1** | 392.602 | 1.234 | 0.436 | 2.828 | 0.005 |
| 240873 | **Tnfsf18** | 38.417 | 1.217 | 0.484 | 2.514 | 0.012 |
| 22361 | **Vnn1** | 74.679 | 1.083 | 0.439 | 2.470 | 0.014 |
| 14727 | **Lilr4b** | 3688.861 | 1.075 | 0.455 | 2.361 | 0.018 |
| 14728 | **Lilrb4a** | 2009.101 | 1.175 | 0.513 | 2.288 | 0.022 |
| 20739 | **Spta1** | 3.910 | 3.415 | 1.525 | 2.239 | 0.025 |
| 16176 | **Il1b** | 4970.257 | 0.915 | 0.412 | 2.222 | 0.026 |
| 19260 | **Ptpn22** | 176.583 | 0.658 | 0.300 | 2.191 | 0.028 |
| 12519 | **Cd80** | 177.742 | 0.877 | 0.401 | 2.188 | 0.029 |
| 243910 | **Nfkbid** | 299.450 | 0.760 | 0.347 | 2.188 | 0.029 |
| 22287 | **Scgb1a1** | 40.530 | 1.084 | 0.501 | 2.164 | 0.030 |
| 170780 | **Cd209e** | 1287.940 | 0.663 | 0.332 | 1.998 | 0.046 |
| 17242 | **Mdk** | 59.511 | 1.032 | 0.525 | 1.966 | 0.049 |
| 278180 | **Vsig4** | 13.448 | 1.332 | 0.689 | 1.934 | 0.053 |
| 20533 | Slc4a1 | 8.753 | 1.715 | 0.919 | 1.865 | 0.062 |
| 11847 | Arg2 | 182.544 | 0.713 | 0.401 | 1.779 | 0.075 |
| 18106 | Cd244a | 268.936 | 0.682 | 0.392 | 1.737 | 0.082 |
| 20296 | Ccl2 | 1116.434 | 0.762 | 0.441 | 1.729 | 0.084 |
| 208154 | Btla | 110.692 | 0.750 | 0.436 | 1.721 | 0.085 |
| 245126 | Tarm1 | 206.000 | 0.726 | 0.435 | 1.668 | 0.095 |
| 170779 | Cd209d | 1875.217 | 0.482 | 0.298 | 1.615 | 0.106 |
| 16160 | Il12b | 4.456 | 1.837 | 1.149 | 1.598 | 0.110 |
| 216799 | Nlrp3 | 711.765 | 0.737 | 0.477 | 1.543 | 0.123 |
| 58205 | Pdcd1lg2 | 57.726 | 0.583 | 0.385 | 1.516 | 0.129 |
| 12524 | Cd86 | 456.530 | 0.498 | 0.347 | 1.436 | 0.151 |
| 16408 | Itgal | 735.433 | 0.501 | 0.360 | 1.392 | 0.164 |
| 170786 | Cd209a | 1695.679 | 0.371 | 0.268 | 1.388 | 0.165 |
| 12522 | Cd83 | 207.437 | 0.394 | 0.287 | 1.373 | 0.170 |
| 193385 | Ripor2 | 479.900 | 0.517 | 0.378 | 1.365 | 0.172 |
| 12484 | Cd24a | 3932.552 | 0.335 | 0.246 | 1.364 | 0.173 |
| 14102 | Fas | 472.058 | 0.434 | 0.319 | 1.360 | 0.174 |
| 12142 | Prdm1 | 826.735 | 0.400 | 0.300 | 1.335 | 0.182 |
| 16797 | Lat | 108.921 | 0.449 | 0.340 | 1.323 | 0.186 |
| 57438 | 7-Mar | 2329.350 | 0.472 | 0.364 | 1.295 | 0.195 |
| 223601 | Fam49b | 1614.754 | 0.490 | 0.379 | 1.292 | 0.196 |
| 30936 | Slc46a2 | 895.028 | 0.443 | 0.345 | 1.285 | 0.199 |
| 16193 | Il6 | 204.086 | 0.773 | 0.607 | 1.275 | 0.202 |
| 12504 | Cd4 | 137.032 | 0.402 | 0.319 | 1.260 | 0.208 |
| .... |  |  |  |  |  |  |

**Table S4:** Overrepresented core enriched genes in mouse skin wounds 1 day post traumatic brain injury as assessed by gene set enrichment analysis of T cell activation Gene Ontology term.
