# Supplementary material for: Traumatic Brain Injury Induces Early Barrier Protective Responses in Incisional Skin Wounds Accelerating Cutaneous Wound Healing
Source: Wound Repair Regen. 2025 Aug 29;33(5):e70079. doi: 10.1111/wrr.70079 (PMC12395893; doi:10.1111/wrr.70079)
Supplement: Supplementary file 7 — Table S5: Overrepresented core enriched genes in mouse skin wounds 1 day post traumatic brain injury as assessed by gene set enrichment analysis of antimicrobial humoral response Gene Ontology term. [file WRR-33-0-s009.docx]

| **Entrez_id** | **Gene_name** | **Base mean** | **Log2 fold change** | **lfcSE** | **Stat** | **p value** |
| --- | --- | --- | --- | --- | --- | --- |
| 55985 | **Cxcl13** | 518.538 | 0.952 | 0.315 | 3.022 | 0.003 |
| 57349 | **Ppbp** | 272.178 | 1.295 | 0.473 | 2.737 | 0.006 |
| 14825 | **Cxcl1** | 485.782 | 0.920 | 0.414 | 2.223 | 0.026 |
| 20311 | **Cxcl5** | 2417.318 | 0.983 | 0.477 | 2.060 | 0.039 |
| 330122 | **Cxcl3** | 650.633 | 1.075 | 0.538 | 1.996 | 0.046 |
| 21946 | **Pglyrp1** | 290.553 | 1.924 | 0.996 | 1.931 | 0.053 |
| 20299 | **Ccl22** | 280.987 | 0.849 | 0.447 | 1.902 | 0.057 |
| 20310 | Cxcl2 | 1747.979 | 0.889 | 0.497 | 1.790 | 0.074 |
| 109820 | Pgc | 10.964 | 4.015 | 2.358 | 1.703 | 0.089 |
| 20295 | Ccl17 | 24.527 | 1.352 | 0.822 | 1.644 | 0.100 |
| 16365 | Acod1 | 2172.115 | 0.646 | 0.435 | 1.484 | 0.138 |
| 546038 | Spag11b | 10.943 | 1.078 | 0.744 | 1.449 | 0.147 |
| 11535 | Adm | 685.315 | 0.588 | 0.413 | 1.423 | 0.155 |
| 17329 | Cxcl9 | 61.390 | 0.935 | 0.667 | 1.401 | 0.161 |
| 100034251 | Wfdc17 | 1212.210 | 0.391 | 0.302 | 1.296 | 0.195 |
| 66438 | Hamp2 | 4.513 | 1.336 | 1.068 | 1.251 | 0.211 |
| 20297 | Ccl20 | 81.220 | 0.589 | 0.471 | 1.251 | 0.211 |
| 20202 | S100a9 | 5443.528 | 0.592 | 0.476 | 1.244 | 0.213 |
| 109648 | Npy | 6.147 | 1.219 | 0.989 | 1.232 | 0.218 |
| 56838 | Ccl28 | 23.189 | 0.695 | 0.585 | 1.190 | 0.234 |
| 209232 | Wfdc5 | 707.816 | 0.367 | 0.322 | 1.140 | 0.254 |
| 20568 | Slpi | 1234.159 | 0.478 | 0.439 | 1.089 | 0.276 |
| 385643 | Kng2 | 65.422 | 0.430 | 0.396 | 1.087 | 0.277 |
| 192200 | Wfdc12 | 84.560 | 0.544 | 0.518 | 1.050 | 0.294 |
| 59032 | Ppp2r3c | 490.130 | 0.297 | 0.304 | 0.978 | 0.328 |
| 12051 | Bcl3 | 878.435 | 0.333 | 0.346 | 0.963 | 0.335 |
| 20301 | Ccl27a | 871.167 | 0.255 | 0.293 | 0.870 | 0.384 |
| 11731 | Ang2 | 9.257 | 0.703 | 0.824 | 0.854 | 0.393 |
| 20292 | Ccl11 | 829.966 | 0.292 | 0.349 | 0.837 | 0.403 |
| 16855 | Lgals4 | 243.901 | 0.303 | 0.372 | 0.814 | 0.416 |
| 20290 | Ccl1 | 9.020 | 0.657 | 0.817 | 0.803 | 0.422 |

**Table S5:** Overrepresented core enriched genes in mouse skin wounds 1 day post traumatic brain injury as assessed by gene set enrichment analysis of antimicrobial humoral response Gene Ontology term.
