# Supplementary material for: Traumatic Brain Injury Induces Early Barrier Protective Responses in Incisional Skin Wounds Accelerating Cutaneous Wound Healing
Source: Wound Repair Regen. 2025 Aug 29;33(5):e70079. doi: 10.1111/wrr.70079 (PMC12395893; doi:10.1111/wrr.70079)
Supplement: Supplementary file 8 — Table S6: Overrepresented core enriched genes in mouse skin wounds 1 day post traumatic brain injury as assessed by gene set enrichment analysis of neuroactive ligand‐receptor interactions Gene Ontology term. [file WRR-33-0-s001.docx]

| **Entrez_id** | **Gene_name** | **Base mean** | **Log2 fold change** | **lfcSE** | **Stat** | **p value** |
| --- | --- | --- | --- | --- | --- | --- |
| 14293 | **Fpr1** | 212.200 | 1.470 | 0.502 | 2.929 | 0.003 |
| 14289 | **Fpr2** | 317.320 | 1.083 | 0.466 | 2.327 | 0.020 |
| 110637 | **Grik4** | 5.752 | 2.257 | 1.024 | 2.205 | 0.027 |
| 17199 | **Mc1r** | 2.158 | 4.546 | 2.229 | 2.039 | 0.041 |
| 11435 | **Chrna1** | 118.327 | 0.721 | 0.358 | 2.016 | 0.044 |
| 19217 | **Ptger2** | 89.798 | 0.789 | 0.399 | 1.980 | 0.048 |
| 74191 | P2ry13 | 212.228 | 0.703 | 0.373 | 1.886 | 0.059 |
| 14064 | F2rl2 | 111.430 | 0.641 | 0.343 | 1.866 | 0.062 |
| 19222 | Ptgir | 361.993 | 0.487 | 0.300 | 1.624 | 0.104 |
| 242425 | Gabbr2 | 6.765 | 1.713 | 1.059 | 1.617 | 0.106 |
| 11449 | Chrng | 3.962 | 1.908 | 1.217 | 1.567 | 0.117 |
| 14600 | Ghr | 3741.830 | 0.610 | 0.396 | 1.538 | 0.124 |
| 11443 | Chrnb1 | 190.561 | 0.529 | 0.350 | 1.514 | 0.130 |
| 14811 | Grin2a | 2.163 | 2.503 | 1.692 | 1.479 | 0.139 |
| 14401 | Gabrb2 | 15.074 | 1.127 | 0.769 | 1.465 | 0.143 |
| 14938 | Gzma | 7.700 | 1.364 | 0.946 | 1.442 | 0.149 |
| 93670 | Tac4 | 56.069 | 1.077 | 0.750 | 1.435 | 0.151 |
| 11535 | Adm | 685.315 | 0.588 | 0.413 | 1.423 | 0.155 |
| 14397 | Gabra4 | 40.170 | 0.766 | 0.540 | 1.417 | 0.157 |
| 15558 | Htr2a | 82.797 | 0.669 | 0.477 | 1.403 | 0.161 |
| 14396 | Gabra3 | 98.638 | 0.647 | 0.470 | 1.377 | 0.169 |
| 14799 | Gria1 | 0.950 | 3.361 | 2.472 | 1.359 | 0.174 |
| 13615 | Edn2 | 8.006 | 1.097 | 0.812 | 1.350 | 0.177 |
| 12273 | C5ar1 | 1427.803 | 0.466 | 0.347 | 1.345 | 0.179 |
| 14804 | Grid2 | 2.913 | 1.864 | 1.397 | 1.334 | 0.182 |
| 15466 | Hrh2 | 165.993 | 0.494 | 0.372 | 1.328 | 0.184 |
| 67168 | Lpar6 | 361.361 | 0.461 | 0.348 | 1.324 | 0.186 |
| 64095 | Gpr35 | 755.401 | 0.483 | 0.371 | 1.303 | 0.193 |
| 14408 | Gabrr1 | 4.155 | 1.748 | 1.349 | 1.295 | 0.195 |
| 14802 | Gria4 | 150.172 | 0.684 | 0.543 | 1.259 | 0.208 |
| 11608 | Agtr1b | 0.851 | 3.186 | 2.563 | 1.243 | 0.214 |
| 14654 | Glra1 | 3.672 | 1.496 | 1.213 | 1.234 | 0.217 |
| 109648 | Npy | 6.147 | 1.219 | 0.989 | 1.232 | 0.218 |
| 78826 | P2ry10 | 168.368 | 0.497 | 0.411 | 1.208 | 0.227 |
| 12671 | Chrm3 | 51.946 | 0.594 | 0.493 | 1.204 | 0.228 |
| 17200 | Mc2r | 28.832 | 0.553 | 0.472 | 1.172 | 0.241 |
| 11607 | Agtr1a | 254.408 | 0.351 | 0.308 | 1.138 | 0.255 |
| 22355 | Vipr2 | 40.414 | 0.598 | 0.530 | 1.129 | 0.259 |

**Table S6:** Overrepresented core enriched genes in mouse skin wounds 1 day post traumatic brain injury as assessed by gene set enrichment analysis of neuroactive ligand-receptor interactions Gene Ontology term.
