# Supplementary material for: Traumatic Brain Injury Induces Early Barrier Protective Responses in Incisional Skin Wounds Accelerating Cutaneous Wound Healing
Source: Wound Repair Regen. 2025 Aug 29;33(5):e70079. doi: 10.1111/wrr.70079 (PMC12395893; doi:10.1111/wrr.70079)
Supplement: Supplementary file 9 — Table S7: Overrepresented core enriched genes in mouse skin wounds 1 day post traumatic brain injury as assessed by gene set enrichment analysis of sensory perception of pain Gene Ontology term. [file WRR-33-0-s003.docx]

| **Entrez_id** | **Gene_name** | **Base mean** | **Log2 fold change** | **lfcSE** | **Stat** | **p value** |
| --- | --- | --- | --- | --- | --- | --- |
| 17199 | Mc1r | 2.158 | 4.546 | 2.229 | 2.039 | 0.041 |
| 19225 | Ptgs2 | 972.724 | 0.873 | 0.478 | 1.827 | 0.068 |
| 20296 | Ccl2 | 1116.434 | 0.762 | 0.441 | 1.729 | 0.084 |
| 21898 | Tlr4 | 896.936 | 0.687 | 0.409 | 1.681 | 0.093 |
| 16160 | Il12b | 4.456 | 1.837 | 1.149 | 1.598 | 0.110 |
| 14811 | Grin2a | 2.163 | 2.503 | 1.692 | 1.479 | 0.139 |
| 93670 | Tac4 | 56.069 | 1.077 | 0.750 | 1.435 | 0.151 |
| 66270 | Retreg1 | 1697.187 | 0.374 | 0.262 | 1.426 | 0.154 |
| 15558 | Htr2a | 82.797 | 0.669 | 0.477 | 1.403 | 0.161 |
| 229323 | Gpr171 | 166.963 | 0.527 | 0.377 | 1.398 | 0.162 |
| 21926 | Tnf | 220.601 | 0.595 | 0.457 | 1.300 | 0.193 |
| 20269 | Scn3a | 108.871 | 0.602 | 0.467 | 1.289 | 0.198 |
| 16181 | Il1rn | 3086.300 | 0.387 | 0.303 | 1.278 | 0.201 |
| 16508 | Kcnd2 | 10.723 | 0.851 | 0.786 | 1.082 | 0.279 |
| 18619 | Penk | 1070.082 | 0.353 | 0.329 | 1.073 | 0.283 |
| 15566 | Htr7 | 107.903 | 0.395 | 0.370 | 1.067 | 0.286 |
| 77125 | Il33 | 4399.244 | 0.440 | 0.419 | 1.048 | 0.294 |
| 16175 | Il1a | 151.998 | 0.348 | 0.342 | 1.019 | 0.308 |
| 17380 | Mme | 684.350 | 0.442 | 0.434 | 1.018 | 0.309 |
| 19204 | Ptafr | 1279.173 | 0.340 | 0.335 | 1.015 | 0.310 |
| 18133 | Nov | 182.561 | 0.344 | 0.343 | 1.005 | 0.315 |
| 12767 | Cxcr4 | 588.698 | 0.418 | 0.417 | 1.003 | 0.316 |
| 270190 | Ephb1 | 46.065 | 0.480 | 0.480 | 1.000 | 0.317 |
| 12772 | Ccr2 | 3544.935 | 0.472 | 0.490 | 0.965 | 0.334 |
| 171209 | Asic3 | 8.444 | 0.757 | 0.802 | 0.943 | 0.346 |
| 13618 | Ednrb | 1685.804 | 0.295 | 0.322 | 0.918 | 0.359 |
| 14812 | Grin2b | 3.102 | 1.149 | 1.271 | 0.904 | 0.366 |
| 56183 | Nmu | 10.070 | 2.291 | 2.594 | 0.883 | 0.377 |
| 12802 | Cnr2 | 221.504 | 0.269 | 0.316 | 0.851 | 0.395 |
| 16485 | Kcna1 | 133.138 | 0.321 | 0.386 | 0.830 | 0.406 |

**Table S7:** Overrepresented core enriched genes in mouse skin wounds 1 day post traumatic brain injury as assessed by gene set enrichment analysis of sensory perception of pain Gene Ontology term.
